# Digital anchors vs. human anchors: a study of the effects of credibility endorsement and psychological distance on policy adoption intention

**DOI:** 10.3389/fpsyg.2025.1650691

**Published:** 2025-11-18

**Authors:** Jinghao Chen, Yuqiyu Zeng, Xiaoyu Qiu

**Affiliations:** 1 School of Public Policy and Management, Guangxi University, Nanning, China; 2 Research Center of Regional Social Governance and Innovation, Guangxi University, Nanning, China; 3 China-ASEAN Collaborative Innovation Center for Regional Development, Nanning, China; 4 Division of Public Policy, Hong Kong University of Science and Technology, Kowloon, Hong Kong SAR, China

**Keywords:** digital anchors, anthropomorphism, psychological distance, credibility endorsement, human anchors

## Abstract

**Purpose/significance:**

Faced with the increasing application of digital human technology in the field of public policy dissemination, this study aims to compare the effects of human and digital human anchors. It explores the mechanisms of psychological distance, anthropomorphism, and credibility endorsement to provide empirical guidance for optimizing policy dissemination practices in the digital era.

**Methods/procedures:**

Three online experiments were conducted using a between-group design to systematically manipulate the type of policy dissemination subject (human vs. digital anchors), degree of digital human anthropomorphism (high vs. low), and credibility endorsement (high vs. low). After viewing the policy videos, participants were recruited online to measure their psychological distance and policy-adoption intention. Process macros were used to test mediation and moderation.

**Results/conclusions:**

The study found that (1) human anchors enhance policy adoption intention more than digital anchors; (2) psychological distance plays a partial mediating role; (3) a high degree of anthropomorphism can narrow the gap between the psychological distance of digital and human anchors; and (4) strong credibility endorsement brings highly anthropomorphic digital anchors closer to humans in terms of psychological distance, while low anthropomorphizers are still at a disadvantage. The effect of digital anchors’ policy communication results from complex interactions between subject characteristics, external empowerment, and audience psychology.

## 1 Introduction

In recent years, with the promotion of digitalization at the global level, publications worldwide, such as the United Nations Development Programme’s (UNDP) “Human Development Report 2025: Humans and Possibilities in the Age of Artificial Intelligence,” have further propelled the development of the virtual digital human industry, presenting new challenges for the dissemination of public policy (United Nations Development Programme, 2025). Whether policies are understood and adopted by the public directly determines their implementation effectiveness. Against the backdrop of rapidly evolving digital technology, digital avatar anchors have gradually entered the public policy communication field, emerging as a new type of communicator. However, the public acceptance of digital avatar anchors remains uncertain. Whether their effectiveness differs from that of human anchors and the underlying psychological mechanisms behind such differences require in-depth research (Xue et al., 2022).

The successful implementation of public policies relies not only on information delivery, but also on public trust and emotional resonance (Goren et al., 2022). The anchor type determines social presence and authenticity in the communication process (Ngo, 2025), which serve as crucial external cues for trust formation (Cheng et al., 2024). In addition, psychological distance acts as a key psychological mechanism that influences public attitudes and behavioral intentions (Blauza et al., 2023). Therefore, exploring how these three factors influence policy adoption intention not only holds practical urgency but also provides theoretical support and practical insights for optimizing policy communication strategies.

However, existing research has largely focused on the application of digital avatars in e-commerce and entertainment domains (Hu et al., 2021; Yan et al., 2025; Chang et al., 2025), with limited systematic research on their roles in public policy communication. Merely introducing digital avatars is insufficient for addressing policy communication challenges. The integration of external empowerment with psychological mechanisms to innovate communication pathways remains a critical issue requiring urgent resolution (Gao et al., 2020). Therefore, this study focuses on policy adoption intentions to systematically compare the communication effectiveness of human and digital avatar anchors. It examines the mediating role of psychological distance and the moderating effects of credibility endorsement and personification levels.

Based on the above background and considerations, this study asks the following questions:

*RQ1*: Does communication type significantly influence public policy adoption intentions?

*RQ2*: Does psychological distance mediate the relationship between a communication subject and policy adoption intention?

*RQ3*: Do credibility endorsements and personification levels moderate the influence of communication on public psychological distance?

## 2 Theoretical analysis and research hypotheses

### 2.1 Digital human anchors study

Digital human anchors are virtual entities generated through computer graphics (CG), artificial intelligence (AI), and associated technologies designed to exhibit anthropomorphic characteristics and interactive capabilities (Cui and Liu, 2023). As “mediators” between information sources and audiences, these anchors operate within various digital platforms, including online media. Their behavior can be driven either by real-time human operators or governed by algorithms and predefined scripts (Chakareski et al., 2022; Miao et al., 2022). Typically rendered as 2D or 3D models, they convey information through a combination of visual representations, speech, and nonverbal cues, showcasing progressively more sophisticated capabilities in semantic understanding and emotion simulation (Chakareski et al., 2022; Miao et al., 2022).

The current research on digital human anchors primarily addresses two key dimensions: anthropomorphic representation and social role enactment. Mindful of the “Uncanny Valley effect” and its potential influence on audience acceptance, researchers have proposed specific guidelines for the optimal visual design of digital human anchors. Studies informed by cognitive conflict theory suggest that attributes such as natural skin tones, realistic body proportions, and distinct gender markers are crucial for fostering positive perceptions of digital humans and their portrayed roles (Schwind et al., 2018). Furthermore, empirical investigations have demonstrated that artificial lip or hand movements can significantly increase negative audience reactions. This response aligns with perceptual mismatch theory and underscores the need to refine the naturalness of facial expressions, lip synchronization, and other “high sensitivity deficits” to improve the acceptability of digital human anchors (Wu et al., 2024). Regarding application domains, digital human anchors are commonly categorized into e-commerce, entertainment, and media sectors (Huang and Yu, 2023; Xu, 2023). For instance, in e-commerce, research indicates that digital human anchors may be more effective than their human counterparts in certain contexts, such as promoting specific products (Yan et al., 2025) or conveying discount information (Chang et al., 2025), to stimulate consumer willingness. Within the entertainment sphere, virtual applications such as Replika exemplify how entertainment anchors, leveraging intelligent voice and real-time interaction technologies, can offer users highly immersive conversational experiences that simulate interactions with a real person (Hu et al., 2021; Laestadius et al., 2024).

In summary, the existing research on digital human anchors has yielded initial findings, predominantly within e-commerce and entertainment, highlighting their potential applications and user preferences in these commercial settings. Nevertheless, a significant portion of the literature prioritizes commercial value, leaving the role and impact of digital human anchors in public affairs communication, particularly public policy communication, underexplored. Given that public-policy communication requires high levels of trust and understanding, the comparative efficacy of digital versus human anchors in this domain warrants immediate investigation. To address this gap, the present study, situated within the public-policy communication context, aims to compare the effects of communicator type (digital human vs. human anchor) on policy adoption intentions. Furthermore, it investigates the potential mediating role of psychological distance and the moderating effects of varying credibility endorsements.

### 2.2 The effect of different policy communication subjects on policy adoption intention

Existing studies have suggested that digital avatars may be less effective than human hosts in terms of communication and persuasion. Communication theory identifies social presence as a critical factor shaping communication outcomes. Media with a higher social presence (e.g., live videos) tend to generate stronger emotional connections and behavioral responses from audiences than those with a low presence (Wang et al., 2024). In tandem, perceived authenticity is essential. Research by Nicolette showed that the inherent authenticity of human hosts helps prevent audience rejection, leading to greater acceptance and recognition that virtual avatars cannot fully replicate (Filova et al., 2025). Additionally, source credibility is a key driver of persuasion (Li et al., 2023). Li found that human hosts, viewed as more authoritative and credible, are more effective than virtual avatars in influencing brand purchase intentions. Since public-policy communication similarly depends on source credibility to facilitate information acceptance and behavior change, this study extends this finding to policy adoption by hypothesizing that human presenters have similar advantages in these contexts.

*H1*: Human anchors are more likely than digital anchors to contribute to the public’s willingness to adopt policies.

### 2.3 The mediating role of psychological distance

Psychological distance denotes the perceived separation between an individual’s self and an object (e.g., event, person, message, encompassing). It encompasses dimensions such as temporal, spatial, social, and hypothetical distances. Construal Level Theory (CLT) posits that psychological distance influences not only cognitive construal, but also significantly impacts attitudes and behavioral intentions. Research has demonstrated that psychological distance can modulate trust (Bandara et al., 2021), affect consumer choice (Goodman and Malkoc, 2012), and shape purchase intentions through its effect on information perception (Liu et al., 2021). Within public-policy communication, a reduced psychological distance toward the information source or message is expected to lead to more favorable information processing; heightened perceived relevance; and increased understanding, agreement, and adoption willingness.

Communicator type is a key determinant of perceived psychological distance. Human anchors generally elicit a higher social presence and greater perceived authenticity (Park, 2024) than digital anchors. Consequently, audiences may perceive human anchors as socially nearer and hypothetically more tangible, reducing the overall psychological distance. This study anticipates that the difference in psychological distance, contingent on communicator type, will subsequently influence the willingness to adopt public policy. Thus, psychological distance is proposed to mediate the effect of communicator type on policy adoption.

*H2*: Psychological distance mediates the relationship between the type of communication subject and the willingness to adopt a policy.

### 2.4 The moderating role of anthropomorphism in digital human anchors

With the growing pervasiveness of human-computer interaction (HCI), anthropomorphism, which is defined as the attribution of human-like characteristics, intentions, or behaviors to nonhuman entities, has emerged as a central concept in HCI research.

Extensive research has indicated that enhancing the anthropomorphism of digital entities generally yields positive outcomes. For instance, in marketing, anthropomorphic chatbots, advertisements, and brand mascots have proven effective in increasing consumer purchase intentions, favorable advertising attitudes, and psychological intimacy (Han, 2021; Ko et al., 2022). In HCI and user acceptance studies, highly anthropomorphic digital humans or bots tend to facilitate more efficient interactions (Lv et al., 2023), garner greater user trust and affective acceptance, and promote better understanding, particularly in service recovery contexts (Frazer, 2022).

These positive effects may be partly attributable to the capacity of anthropomorphism to reduce psychological distance. When digital entities exhibit a high degree of similarity to humans in appearance, vocal characteristics, and demeanor (Frazer, 2022), audiences may perceive them as socially “closer” and hypothetically more “real.” These attributes, in turn, can lessen the perceived disconnect that is often experienced in human-machine interactions.

Consequently, we theorize that the degree of anthropomorphism exhibited by digital anchors may modulate the effect of communicator type (human versus digital) on the public’s perceived psychological distance. Based on this reasoning, the following hypothesis is proposed:

*H3*: The degree of anthropomorphism in digital humans modulates the effect of the communication subject type on psychological distance. Specifically, under conditions of low anthropomorphism, the psychological distance between digital human and human anchors is more pronounced; under conditions of high anthropomorphism, this difference diminishes.

### 2.5 The reconditioning role of creditability endorsements

Credibility endorsement is the process of bolstering the perceived credibility of a message or information source through external cues such as endorsements from authoritative entities or evidence of broad social acceptance. This process serves as a key mechanism for effective dissemination of information.

As a significant cognitive heuristic (Mena et al., 2020), credibility endorsement enables audiences to rapidly evaluate source or message credibility, particularly in situations of information overload or uncertainty, by relying on cues such as reputation, consistency, and social approval (Metzger and Flanagin, 2013). The efficacy of this mechanism has been established across diverse domains. However, its impact can vary depending on factors such as product type (e.g., experiential versus search goods) and the nature of the endorser (e.g., platform-based endorsements such as Netflix recommendations versus traditional celebrity endorsements).

Within the framework of this study, we propose that external credibility endorsements exert a second-order moderating influence (or re-moderating effect) on the previously discussed moderating effect of anthropomorphism (i.e., the degree to which anthropomorphism alters the impact of anchor type on psychological distance). The underlying rationale is that the strength of external credibility cues can shift the audience’s reliance away from the communicator’s intrinsic characteristics (such as their human versus non-human nature and degree of anthropomorphism) toward these external signals, thereby influencing the perceived psychological distance.

Therefore, the following hypothesis is proposed.

*H4*: Credibility endorsement affects the influence of anthropomorphism on psychological distance. Specifically, under strong credibility endorsement conditions, the impact of anthropomorphism on psychological distance is weakened; under weak credibility endorsement conditions, the impact of anthropomorphism on psychological distance is enhanced.

## 3 Research design

**Figure 1:**
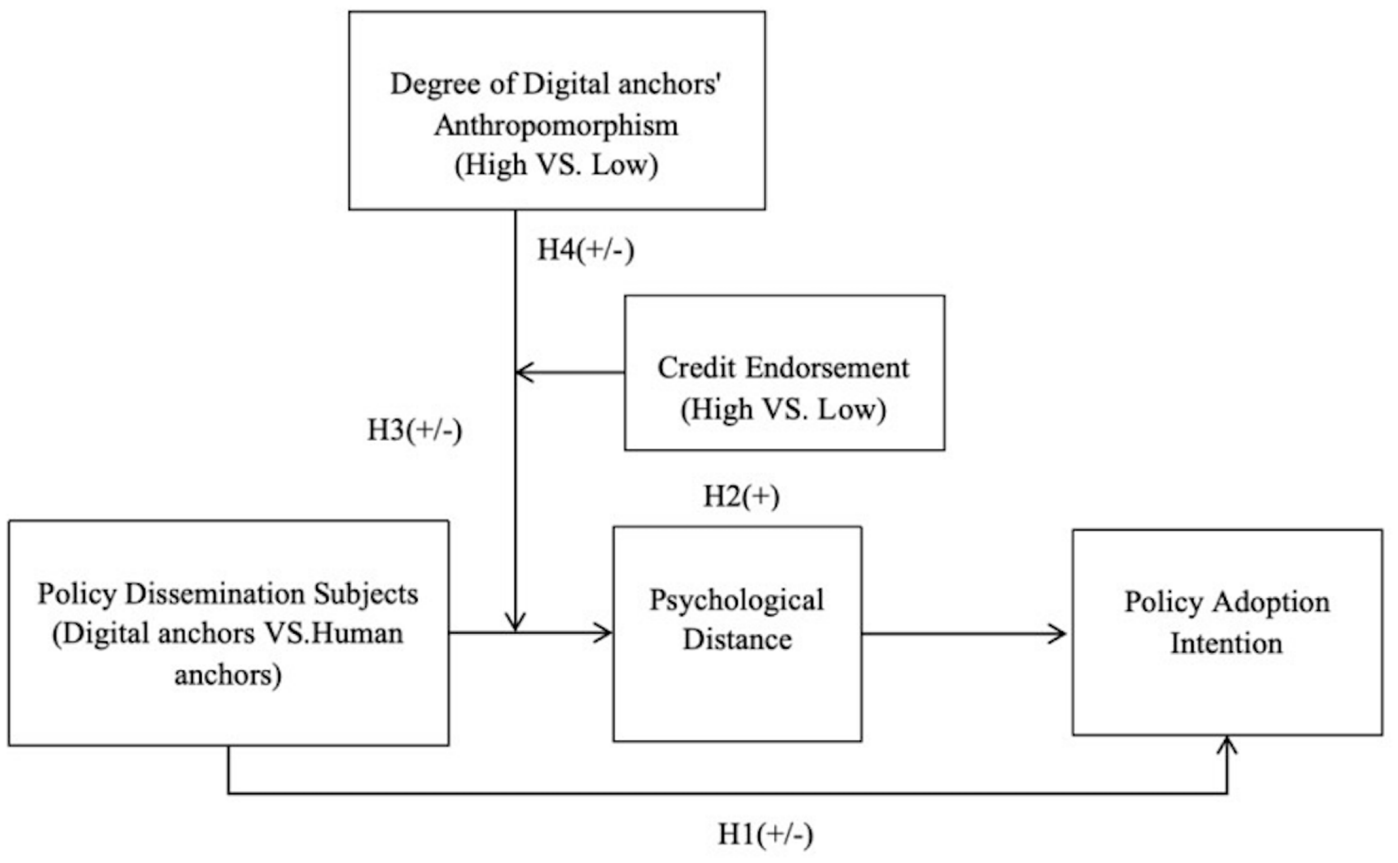
Research framework. ***p < 0.001.

As shown in Figure 1, our research empirically evaluates the proposed hypotheses through three experimental studies. Study 1 provides an initial examination of the effect of different policy communicator types (human vs. digital anchors) on policy adoption intention (H1) and investigates the mediating role of psychological distance in this relationship (H2). Subsequently, Study 2 explores the moderating influence of digital anchors’ anthropomorphism (H3), and Study 3 examines the moderating (or re-moderating) effect of credibility endorsement (H4).

### 3.1 Sample collection and data

To enhance external validity, students were excluded from the formal experiments. This exclusion stems not solely from the “plastic pollution control” topic, but from widespread findings in social science and behavioral research that student groups exhibit high structural homogeneity in age, educational background, and other factors (Gallander Wintre et al., 2001), Their social roles remain fluid, their connection to public policy interests is relatively weak, and their attitudes are more susceptible to fluctuation due to conformity and peer influence (Peterson and Merunka, 2014). These characteristics may weaken the generalizability of the conclusions drawn in this study. Therefore, student samples were excluded from the formal experiment for methodological reasons.

This study employed the professional statistical software G*Power for the preliminary sample size calculation and analysis to ensure the accuracy of the experimental effects and stability of the statistics. Considering a moderate effect size of 0.25, significance level of 0.05, and power of 0.8, studies 1, 2, and 3 were projected to require sample sizes of 128, 158, and 270, respectively. Study 1 collected 128 questionnaires; Study 2 collected 241 questionnaires; and Study 3 collected 470 questionnaires, totaling 839.

Regarding demographic characteristics, the sample exhibited a balanced distribution across sex, age, occupation, and educational background, reflecting the general public profile. This distribution supported the reliability and interpretability of the experimental results (Table 1).

**Table 1:** Demographic data of the study sample.

Variable	Option	Frequency	Percentage (%)
Sex	Male	318	37.9
	Female	521	62.1
Age	21–30 years old	332	39.5
	31–40 years old	400	47.6
	41–50 years old	67	8
	51–60 years old	33	3.9
	Over 60 years old	7	0.8
Occupation Type	State-owned enterprise	106	12.6
	Public institution	94	11.2
	Civil servant	40	4.8
	Private enterprise	539	64.2
	Foreign-funded enterprise	60	7.1
Highest Education	Junior high school	5	0.6
	General high school/technical secondary school/technical school/vocational high school	38	4.5
	Junior college	58	6.9
	Undergraduate	591	70.4
	Master’s degree	139	16.5
	Doctoral degree	8	1

### 3.2 Experimental content design

This study selected “plastic pollution control” as its experimental topic based on three primary considerations: First, plastic pollution represents a quintessential environmental governance issue, involving ecological sustainability, social responsibility, and economic equilibrium. It constitutes a complex policy context characterized by an interdisciplinary nature and the interplay of multiple stakeholders. Compared to topics such as taxation and personal health, plastic pollution control combines public relevance with urgency, making the topic more likely to spark widespread audience attention and discussion. These factors facilitate a better understanding of, and engagement with, the issue across diverse cultural backgrounds. Second, environmental policies are often regarded as “marginal.” During policy adoption, they minimize interference from existing stances and political ideologies, allowing audiences to focus more intently on the policy’s substance and communication methods (Guston, 2001). Therefore, using environmental governance issues as the experimental material ensures that participants from diverse cultural backgrounds engage in discussions within a similar framework, enhancing the contextual relevance of the experiment and the interpretive power of its results. Finally, tackling plastic pollution has become a key priority in China’s governance efforts, exemplified by the issuance of the “Action Plan for Plastic Pollution Control during the 14th Five-Year Plan Period.” This official focus elevates the policy issue to a high practical relevance, provides a concrete policy context for cultural adaptation, and strengthens the real-world significance of this study.

### 3.3 Questionnaire design

To ensure content validity, all scales in this study were designed by referencing established and mature instruments. The research comprises four variables and 14 items using a 7-point Likert scale, as shown in Table 2.

**Table 2:** Variable assessment scale.

Measured variable	Measurement item	Cronbach’s alpha	KMO value	References
Policy Cognition	How well do you understand the policy field mentioned in this study?	0.883	0.801	Rahman et al. (2025)
	Do you usually pay attention to news or information related to this policy field?			
	Without any other information, how high is your support for the government’s introduction of this policy?			
	If this policy is implemented, how likely are you to take practical actions to cooperate soon with this policy?			
Psychological Distance	I can easily absorb and accept the content broadcast by this anchor.	0.950	0.722	Hernández-Ortega (2018)
	The content broadcast by this anchor resonates with me.			
	I think the broadcast of this anchor feels very natural.			
Degree of Anthropomorphism of Digital Human Anchor	I think the performance of this anchor makes me feel professional.	0.899	0.741	Touré-Tillery and McGill (2015), Go and Sundar (2019), and Ollier et al. (2021)
	I think the appearance characteristics of this anchor are similar to those of professional news anchors.			
	I think this anchor matches the policy content introduced.			
Willingness to Adopt Policy	After watching this video, I have a better understanding of this policy.	0.923	0.850	Yuen et al. (2019), Amin et al. (2021), Twum et al. (2021), Chen et al. (2024)
	I believe this policy is beneficial to society.			
	If given the opportunity, I will relay and recommend this policy to others.			
	If this policy is implemented, I am willing to support, abide by, and cooperate with it.			

## 4 Data analysis

### 4.1 Study 1

#### 4.1.1 Experimental design and subjects

Study 1 employed a one-way, two-level (communicator type: digital vs. human anchors) between-subjects experimental design. The primary objectives were to investigate the influence of different communicator types on public policy adoption intentions and examine the mediating role of psychological distance in this relationship. Through the online Credamo platform, 128 participants were recruited.

#### 4.1.2 Experimental procedure and pre-experimentation

Participants were randomly assigned to either a human anchor or a digital human anchor group (details of the experimental stimulus materials are provided in Appendix). They then followed on-screen instructions to complete the experimental procedure sequentially: (1) provision of demographic information (e.g., sex, age); (2) completion of a pre-test survey assessing awareness of the “plastic pollution control” policy; (3) viewing of the experimental video corresponding to their assigned group; and (4) completion of scales measuring psychological distance and policy adoption intention.

To assess the validity of the measurement tools, experimental videos, and procedures, Study 1 conducted a pre-experiment using an online questionnaire distributed through a link prior to the formal experiment. A total of 123 questionnaires were collected, of which 90 were deemed valid, ensuring clarity of the experimental procedures and item wording.

#### 4.1.3 Discussion of data analysis and hypothesis testing results

*Manipulation test*. The 95% confidence intervals for changes in the subjects’participants’ policy attitudes and willingness to adopt scores for the same policy between the pre-test and post-test questionnaires were compared using *t*-tests to conduct the manipulation test for Study 1. The results showed that the change in policy adoption intention scores of the experimental group that watched the experimental material was significant compared with the control group that did not watch the experimental video (M _policy awareness_ = 4.76, SD = 1.21, M _policy adoption_ = 5.05, SD = 1.00). The findings suggest that the policy propaganda news received from the different policy dissemination subjects was persuasive, and that the audience’s willingness to adopt the policy was stronger afterwards, indicating that the news received from different subjects is compelling. The audience’s desire to adopt the policy became stronger after viewing the experimental materials, confirming the validity of the experimental manipulation.

**Figure 2:**
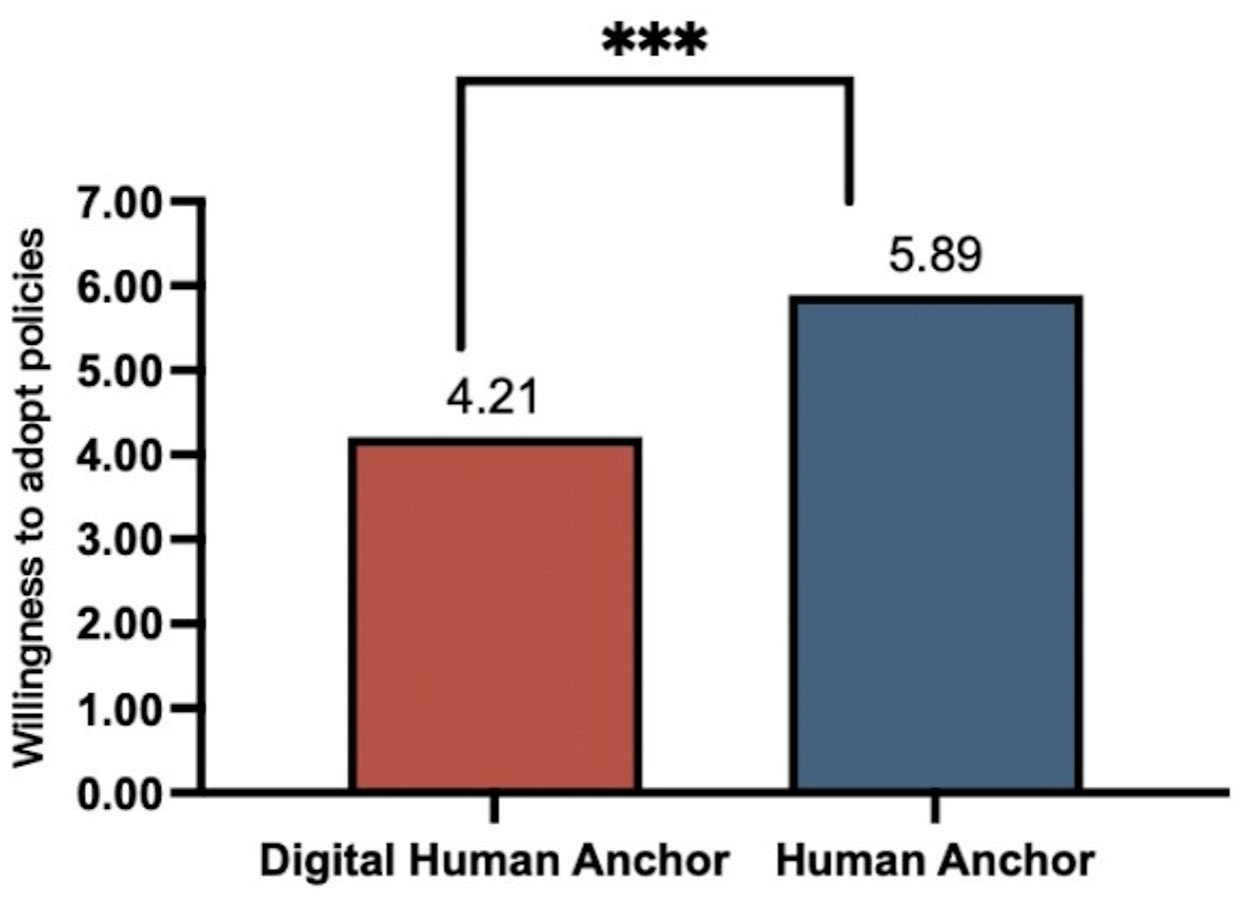
The effect of different policy communication subjects on policy adoption intention. ***p < 0.001.

Main effect of anchor type on policy adoption intention. An independent samples *t*-test was used to compare policy adoption intentions between participants exposed to human or digital anchors. The analysis revealed that participants in the human anchor group exhibited significantly greater policy adoption intentions (M _human anchor_ 5.89, SD = 0.59) than those in the digital anchor group (M _digital anchors_ = 4.21, SD = 0.53; t (126) = 16.924, *p <* 0.001). This result provides robust support for H1, as shown in Figure 2.

*Mediation analysis*. To test the mediating role of psychological distance (H2), a bootstrapping procedure (PROCESS macro, Model 4; 5,000 samples; 95% confidence interval) was employed, with the anchor type as the independent variable and policy adoption intention as the dependent variable. The results indicate a significant indirect effect of anchor type on policy-adoption intention via psychological distance (effect = 1.0889; 95% CI [0.7108, 1.6781]), as the confidence interval did not include zero. The total effect of anchor type on policy adoption intention was also significant (effect = 1.6719, t = 16.9236, *p <* 0.001). After accounting for the mediator (psychological distance), the direct effect of anchor type on policy adoption intention remained significant (effect = 0.5830; t = 3.3204, *p <* 0.001). The results of this analysis are presented in Table 3. These findings demonstrate that psychological distance partially mediates the relationship between anchor type and policy-adoption intention, thus supporting H2.

**Table 3:** Modeling test of the mediating effect of psychological distance (*N* = 128).

Form	Effect	SE	*t*	*P*	95% CI		Reach a verdict
					LLCI	ULCI	
Aggregate effect	1.6719	0.0988	16.9236	0.000***	1.4764	1.8674	Intermediary
Direct effect	0.5830	0.1756	3.3204	0.000***	0.2355	0.9304	
Intermediary effect	1.0889	0.2490	–	Does not contain 0	0.7108	1.6781	

***P < 0.001.

#### 4.1.4 Discussion of results

The findings from Study 1 offer initial evidence that, for identical policy news content, human anchors are more effective than digital anchors in fostering audience policy support; this outcome supports H1. Furthermore, psychological distance was identified as a mediator of this relationship, supporting H2.

### 4.2 Study 2

#### 4.2.1 Experimental design and purpose

In this study’s between-group experimental design, participants were randomly divided into three groups of 2 (policy dissemination subject: digital anchors vs. human anchor) × 2 (digital anchor anthropomorphism: high vs. low). The intent was to explore the moderating role of digital human anchor anthropomorphism on the relationship between policy dissemination and willingness to adopt policy.

#### 4.2.2 Experimental manipulation and pre-experimentation

For Study 2, three distinct experimental videos featuring policy interpretation content were used to maintain content consistency with Study 1 (stimulus details are provided in Appendix). Building on Study 1, the videos featured (1) a human anchor, (2) a 2D low-anthropomorphism digital anchor, and (3) a newly developed 3D high-anthropomorphism digital anchor (sourced from the Arigato AIGC platform). This approach was designed to create distinct conditions based on the visual representation and movement characteristics of the anchor, specifically differentiating between human, low-anthropomorphism digital, and high-anthropomorphism digital anchors.

A pre-test involving 129 participants was conducted to validate the manipulation of the digital anchor anthropomorphism. After viewing one of the experimental videos, participants rated the perceived anthropomorphism of the digital anchors using a scale (*α* = 0.705) adapted from the Degree of Robot Anthropomorphism Scale by Ollier et al. (2021), Maferima (2015), and Go and Sundar (2019). The scale was used to assess the anchor’s professionalism, resemblance to a professional news anchor, and perceived fit with the policy content.

One-way ANOVA revealed significant differences in perceived anthropomorphism across the three anchor types. *Post hoc* tests indicated that the human anchor was rated the highest (M _human anchor_ = 5.32). Crucially, the high-anthropomorphism digital anchor (M _high-anthropomorphism digital human anchor_ = 4.76, SD = 0.629) was rated as significantly more anthropomorphic than the low-anthropomorphism digital anchor (M _low-anthropomorphism digital anchor_ = 3.93, SD = 0.748, *p <* 0.001). This result confirms the successful manipulation of the anthropomorphism levels for digital human anchors.

#### 4.2.3 Experimental procedure and measurements

For the main experiment of Study 2, 241 participants were randomly assigned to one of the three experimental groups (human anchors, high-anthropomorphism digital human anchors, or low-anthropomorphic digital human anchors). The experimental procedure mirrored that of Study 1, except for a key difference being: the specific anchor type presented in the stimulus video, which reflects the manipulation of the digital anchors’ anthropomorphism. All measurement scales, including those for prior policy knowledge and attitudes (*α* = 0.927), psychological distance (*α* = 0.920), and policy adoption intention (*α* = 0.892), were identical to those employed in Study 1.

#### 4.2.4 Data analysis and hypothesis testing

*Manipulation test*. A paired samples *t*-test was used to compare the *a priori* knowledge of policy in the pre-test questionnaire and the willingness to adopt policy in the post-test questionnaire. The results showed that the audience could adapt to the experimental situation well. Cognition and willingness to adopt the policy (*p <* 0.001) significantly increased, and the manipulation of the experimental material was effective. The results of the independent samples *t*-test showed a significant difference in anthropomorphizing degree among different groups [*t*(239) = −5.467, *p <* 0.001], and the anthropomorphizing degree of the high-anthropomorphism digital anchor was significantly higher than that of the low-anthropomorphism digital anchor (M _high-anthropomorphism digital anchor_ = 5.05, SD = 0.953 vs. M _low-anthropomorphism digital anchor_ = 4.12, SD = 1.108; *p <* 0.001). Thus, the degree of anthropomorphizing of digital anchors was successfully manipulated.

Main effect of communicator type on policy adoption intention. A one-way ANOVA was conducted to examine the impact of anchor type (human, low-anthropomorphism digital, and high-anthropomorphism digital anchor) on policy adoption intentions. The analysis revealed a significant overall effect of communicator type [*F*(2, 238) = 306.442, *p <* 0.001]. The mean policy adoption intentions were highest for the human anchor (M _human anchor_ = 6.09), followed by the high-anthropomorphism digital anchor (M _high-anthropomorphism digital anchor_ = 5.32), and lowest for the low-anthropomorphism digital anchor (M _low-anthropomorphism digital anchor_ = 3.92). These results further support Hypothesis H1.

The mediating role of psychological distance in the relationship between communicator type and policy-adoption intention was re-examined using Hayes’ PROCESS macro (Model 4; 5,000 bootstrap samples; 95% confidence intervals). The results indicated that the mediating effect of psychological distance was significant (LLCI = 0.1354, ULCI = 0.4437, excluding 0), with a mediating effect value of 0.2824. This result demonstrates that policy communicators exert a significant influence on policy adoption intentions through psychological distancing. Furthermore, after controlling for psychological distance (treated as a fixed mediating variable in the model), the direct effect of policy communicators on policy adoption intention remained significant (LLCI = 1.0678, ULCI = 1.3697, excluding 0). This finding confirms that psychological distance partially mediates the relationship between policy communicators and policy adoption intention, thereby providing further support for H2.

**Figure 3:**
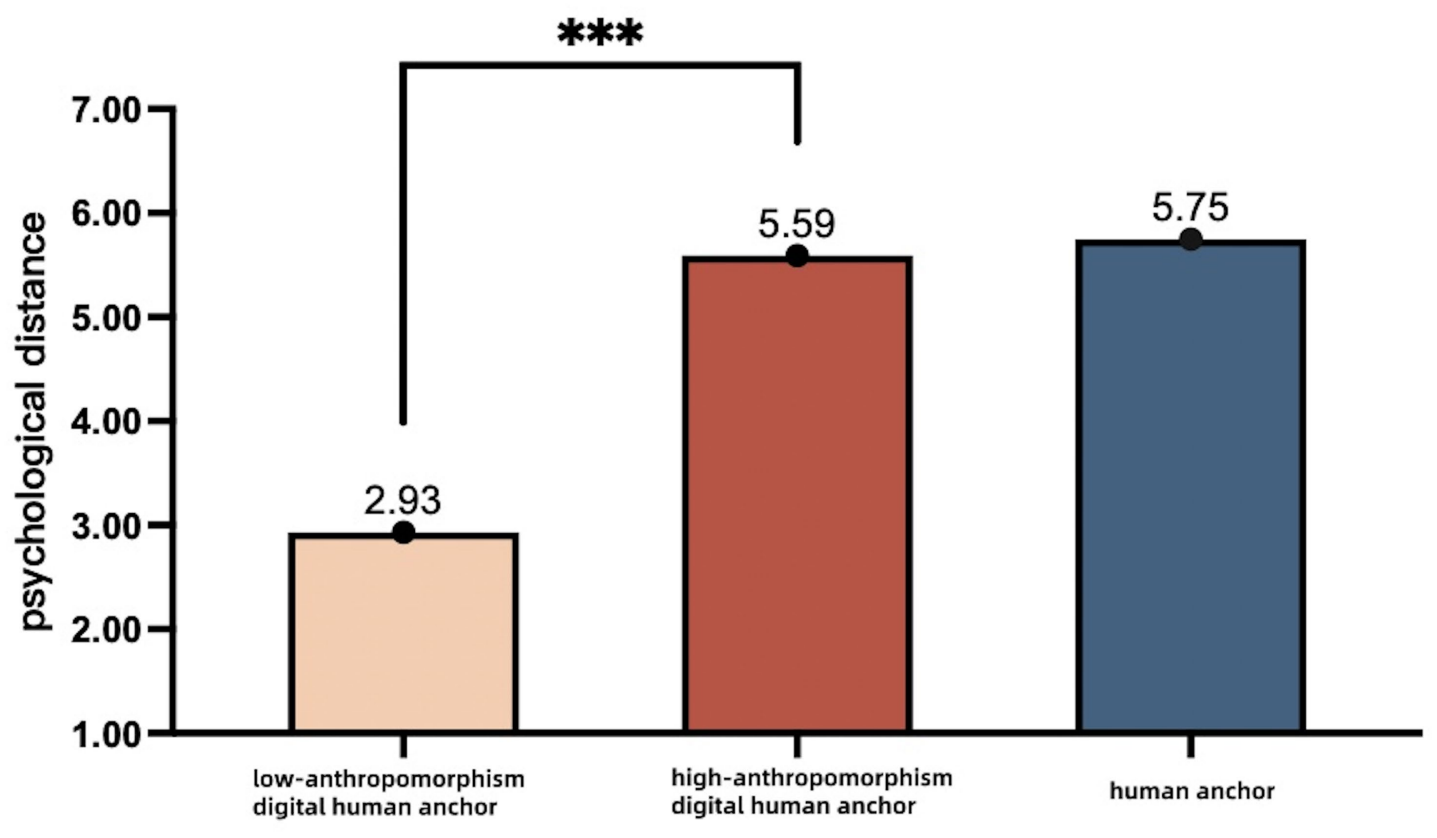
Comparison of mean psychological distance in the interaction between subject of policy communication and degree of anthropomorphism. ***p < 0.001.

Moderating effects test. Two-way ANOVA was used to analyze the moderating effect of the degree of anthropomorphism on policy dissemination. The results showed a significant main effect of policy dissemination [*F*(1, 237) = 238.835, *p <* 0.001], degree of anthropomorphizing [*F*(1, 237) = 2210.094, *p <* 0.001], and the interaction between the two [*F*(1, 237) = 300.325, *p <* 0.001]. Specifically, in the low anthropomorphizing level context, the high-anthropomorphism digital anchor group was closer psychologically [(M _high-anthropomorphism digital anchor_ = 5.59, SD = 0.96), *F*(1, 237) = 330.95, *p <* 0.001] compared with the low-anthropomorphism digital anchor group (M _low-anthropomorphism digital anchor_ = 2.93, SD = 0.83). However, in the high anthropomorphizing level context, no significant difference in psychological distance was found between the high-anthropomorphism digital anchor group (M _high-anthropomorphism digital anchor_ = 5.59, SD = 0.96) and the human anchor group (M _human anchor_ = 5.75, SD = 0.95 [*F*(1, 237) = 1.34, *p* > 0.05]. The results of this analysis are presented in Table 4 and Figure 3.

**Table 4:** The interaction between the subject of policy communication and the degree of anthropomorphizing.

Source of variation	Sum of squares	df	Mean square	*F*	*p*	Partial Eta-square (Partial *η*^2^)
Intercept	5450.203	1	5450.203	6367.374	0.000***	0.964
Policy dissemination subjects	204.433	1	204.433	238.835	0.000***	0.501
Degree of anthropomorphizing of digital human anchors	1891.747	1	1891.747	2210.094	0.000***	0.903
Policy dissemination subject* degree of anthropomorphizing of digital human anchors	257.066	1	257.066	300.325	0.000***	0.558
Residual	203.718	238	0.856			

R^2^ = 0.660, ***P < 0.001.

#### 4.2.5 Discussion of results

Study 2 further validated the relationships among communicator type, psychological distance, and policy adoption intention, thereby corroborating the findings of Study 1 (H1 and H2). Study 2 tested and confirmed the moderating effect of digital anchor anthropomorphism (H3). The results demonstrate that the degree of digital human anthropomorphism moderates the impact of anchor type on perceived psychological distance. Specifically, the human anchor is perceived as psychologically closer than a digital anchor with low anthropomorphism. However, this difference in perceived psychological distance was not evident when comparing the human anchor to the high-anthropomorphism digital anchor, thus supporting H3.

### 4.3 Study 3

#### 4.3.1 Experimental design and purpose

Study 3 employed a between-subjects experimental design with three factors: communicator type (human vs. digital anchors), digital anchor anthropomorphism (high vs. low), and credibility endorsement level (high vs. low). This study aimed to investigate the three-way interaction among these factors on perceived psychological distance, specifically examining how credibility endorsement levels moderate the interplay between communicator types and digital anchors’ anthropomorphism.

#### 4.3.2 Experimental manipulation and pre-experimentation

A pre-experimental phase was conducted to perform manipulation checks on the (1) effectiveness of the experimental materials, (2) perceived anthropomorphism of the digital anchors, and (3) efficacy of the credibility endorsements.

Results from a paired-samples *t*-test indicated a significant change in the participants’ policy-related assessments from pre-exposure to post-exposure to the experimental materials (*p <* 0.01). This outcome confirms the effectiveness of the experimental stimuli in influencing policy perceptions.

The manipulation of the digital anchor anthropomorphism followed the procedure used in Study 2. A separate sample of 205 participants rated the perceived anthropomorphism of the digital anchors presented in the experimental videos using the same measures as in Study 2. An independent sample *t*-test confirmed that the high-anthropomorphism digital anchor was perceived as significantly more anthropomorphic (M _high-anthropomorphism digital anchor_ = 5.01, SD = 0.63) than the low-anthropomorphism one (M _low-anthropomorphism digital anchor_ = 3.49, SD = 0.61; *p <* 0.001). This result confirms the successful manipulation of the anthropomorphism levels of the digital anchors.

For the credibility endorsement manipulation check, 205 participants were randomly assigned to groups varying in credibility endorsement levels (high vs. low) associated with the experimental videos (stimulus details in Appendix). After exposure, participants rated the perceived credibility of the endorsement/source. An independent samples *t*-test indicated that stimuli with high-credibility endorsements were perceived as significantly more credible than those with low-credibility endorsements (*p <* 0.05). This result confirms the successful manipulation of the credibility endorsement levels.

#### 4.3.3 Experimental procedure and measurements

In Study 3, 470 participants were recruited for the main experiment and randomly assigned to one of six groups with experimental conditions reflective of a 2 (communicator type: human vs. digital anchors) × 2 (digital anchor anthropomorphism: high vs. low) × 2 (credibility endorsement: high vs. low) between-subjects design framework. The experimental procedure largely mirrored those of Studies 1 and 2, except for the inclusion of credibility endorsement manipulations alongside anchor types and (for digital anchors) anthropomorphism manipulations. The scales for prior policy knowledge and attitudes (*α* = 0.883), psychological distance (*α* = 0.950), and policy adoption intention (*α* = 0.923) were identical to those used in Study 1. The manipulation check measures for digital anchor anthropomorphism (*α* = 0.899) and credibility endorsement were consistent with those validated in the pre-experimental phase.

#### 4.3.4 Data analysis and hypothesis testing

*Manipulation tests*. A paired-sample *t*-test was conducted on policy cognition and the willingness to adopt policies. The results showed that audience members were able to substitute for themselves in the experimental situation (*p <* 0.001). After watching the video, the willingness to adopt the policy in the post-test questionnaire improved significantly and positively compared to the policy cognition and attitude in the pre-test questionnaire (M _Policy Adoption_ = 6.05 > M _Policy Cognition_ = 4.97); thus, the experimental material’s manipulation was valid. The results of the independent samples t-test showed that the perceived level of anthropomorphism of subjects in the high-fit group was significantly higher than that of subjects in the low-fit group (M _high-anthropomorphism digital anchor_ = 5.14, SD = 1.46 vs. M _low-anthropomorphism digital anchor_ = 4.22, SD = 0.85; t = −6.849, *p <* 0.05). The manipulation of the anthropomorphism of digital human anchors was successful. Finally, we tested whether the audience could accurately determine the credibility of the video based on the type of endorsement. To verify whether participants could accurately distinguish between high- and low-credibility endorsements, 470 of them were randomly assigned to two types of videos: high-credibility and low-credibility endorsements. After the participants watched the experimental videos, they completed the relevant scales. The results showed a significant difference between high- and low-credibility endorsements in the completion of the psychological distance questionnaire (M _low credibility endorsement_ = 4.07 < M _high credibility endorsement_ = 4.87, t = −5.247, *p <* 0.001) and the completion of the willingness to adopt policies questionnaire (M _low credibility endorsement_ = 5.30 < M _high credibility endorsement_ = 5.86, t = −5.046, *p <* 0.001). These findings suggest that credibility endorsement was manipulated successfully at both the high and low levels.

**Figure 4:**
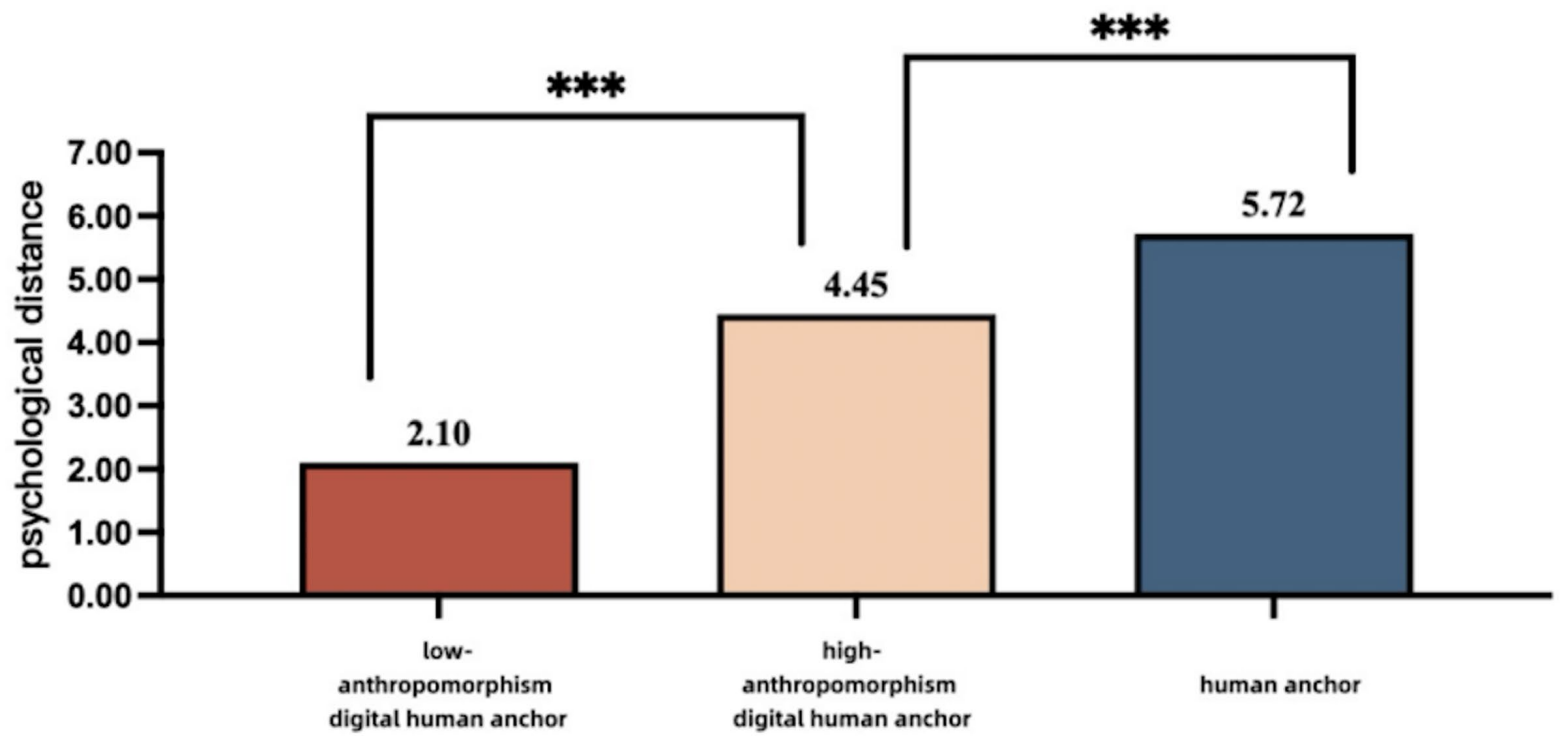
The influence of policy communication subjects and the personification degree of digital hosts on psychological distance under low credibility endorsement.

**Figure 5:**
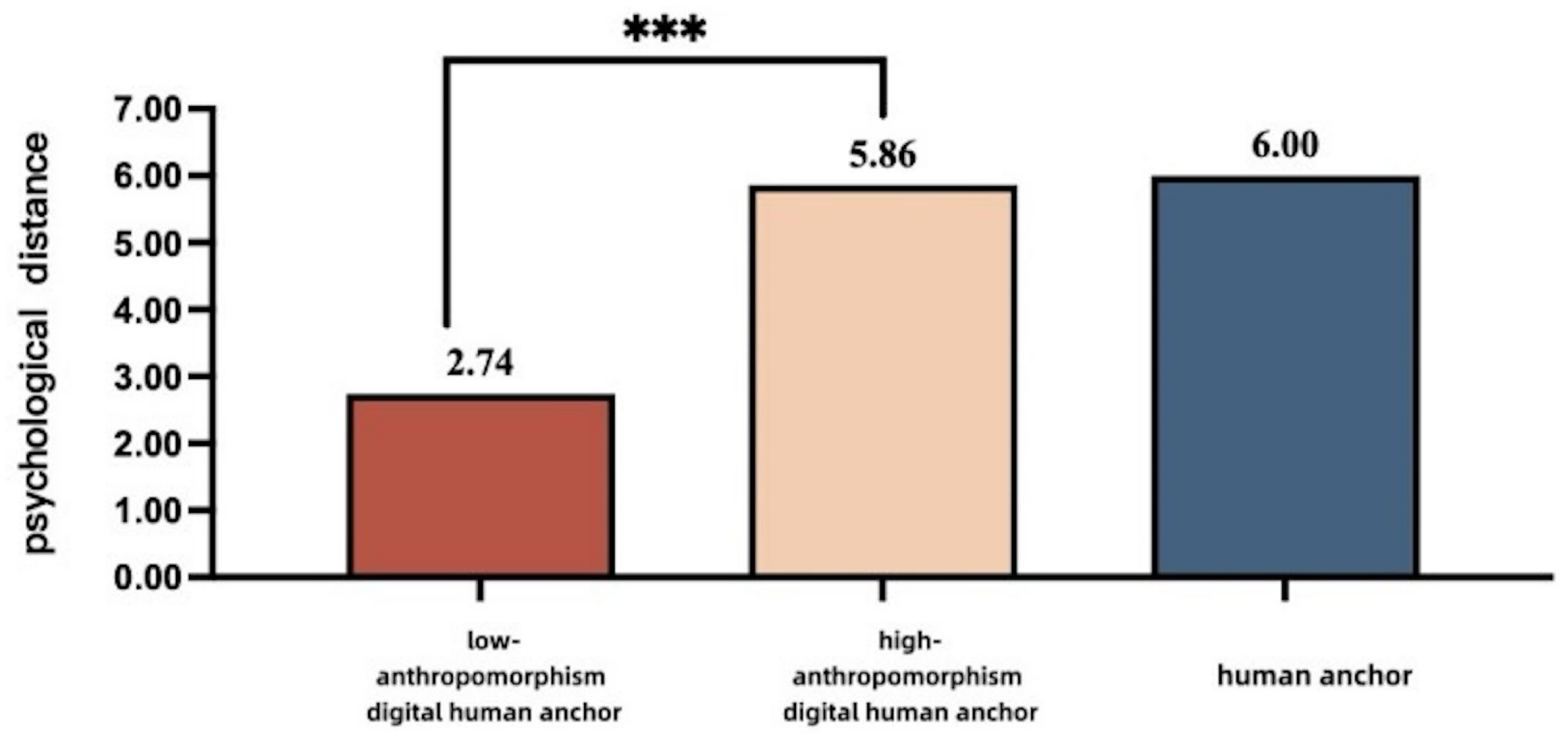
The influence of policy communication subjects and anthropomorphism of digital human anchors on psychological distance under high credibility endorsement.

Moderating effect test. We used a three-way ANOVA to test the moderating effect of credibility endorsement on the moderating effect of anthropomorphism. The results showed a significant main effect of policy dissemination subject [*F*(1, 462) = 84.624, *p <* 0.001], a significant main effect of digital human anchors anthropomorphizing level [*F*(1, 462) = 1261.965 *p <* 0.001], and a significant main effect of credibility endorsement [*F*(1, 462) = 153.425 *p <* 0.001]. More importantly, the interaction between the policy dissemination subject, degree of anthropomorphism of the digital human anchors, and credibility endorsement on the public’s psychological distance was significant [*F*(1, 462) = 27.992, *p <* 0.001]. Simple effect results showed that in the low credibility endorsement context, the psychological distance of participants in the low-anthropomorphism digital anchor group was significantly lower than that of the high-anthropomorphism digital anchor [*F*(1,462) = 469.927, *p <* 0.001; M _low-anthropomorphism digital anchor_ = 2.10, SD = 0.076; M _high-anthropomorphism digital anchor_ = 4.45, SD = 0.077], and that the human anchor group’s psychological distance, on the other hand, was significantly higher than in the high-anthropomorphism digital anchor subject group [*F*(1,462) = 135.936, *p <* 0.001; M _high-anthropomorphism digital anchor_ = 4.45, SD = 0.077; M _human anchor_ = 5.72, SD = 0.077]. The psychological distance of participants in the high-anthropomorphism digital anchor group was significantly higher than that of participants in the low-anthropomorphism digital anchor group in the high-credibility endorsement context [*F*(1,462) = 814.517, *p <* 0.001; M _high-anthropomorphism digital anchor_ = 5.86, SD = 0.077; M _low-anthropomorphism digital anchor_ = 2.74, SD = 0.077]. However, that of the human anchor was similar to that of the high-anthropomorphism digital anchor in the high-credibility endorsement context and was not significantly different [*F*(1,462) = 1.736, *p* > 0.05; M _high-anthropomorphism digital anchor_ = 5.86, SD = 0.077; M _human anchor_ = 6.00, SD = 0.076]. In summary, the test results indicate that credibility endorsement, as an important external cue, significantly moderates the degree to which anthropomorphism influences the process of the anchor type affecting psychological distance. The results of the analysis are shown in Table 5 and Figures 4, 5.

**Table 5:** Three-way interaction of policy dissemination subject with degree of anthropomorphizing and credibility endorsement.

Source of variation	Sum of squares	df	Mean square	*F*	*P*	Partial Eta Square (Partial *η*^2^)
Intercept	7474.055	1	7474.055	16206.528	0.000***	0.972
Policy dissemination subjects	39.026	1	39.026	84.624	0.000***	0.154
Degree of anthropomorphizing of digital human anchors	581.987	1	581.987	1261.965	0.000***	0.731
Credit endorsement	70.756	1	70.756	153.425	0.000***	0.248
Policy dissemination subject * degree of anthropomorphizing of digital human anchors * credibility endorsement	25.818	2	12.909	27.992	0.000***	0.108
Residual	213.985	464	0.461			

R^2^ = 0.842, ***P < 0.001.

#### 4.3.5 Discussion of results

Study 3 verified that the interaction effects of the degree of anthropomorphism of the policy dissemination subject and digital human anchors on the public’s psychological distance differed significantly across credibility endorsement scenarios, and the findings support H4. Specifically, in the low-credibility endorsement scenario, the human anchor was closest to the public compared to the digital anchors, whereas the low-anthropomorphism digital anchor was the furthest away from the audience. In contrast, in the high-credibility endorsement scenario, human anchors still have a closer psychological distance compared with that of the high-anthropomorphism digital anchor, but the difference between the two is further minimized, and both have a closer psychological distance to the audience than the low-anthropomorphism digital anchor.

## 5 Research findings and contributions

### 5.1 Conclusions of the study

This study systematically investigates the efficacy of emerging digital anchors in public policy communication by examining the impact of the communicator type on public policy adoption intentions. It further analyzes the complex interplay among psychological distance, anthropomorphism, and credibility endorsement. Based on the empirical findings, the primary conclusions are as follows.

First, human anchors demonstrate a significant advantage in fostering policy adoption, with psychological distance emerging as a critical mediating mechanism. Second, the effectiveness of digital anchors is contingent on context, with the degree of anthropomorphism acting as a significant moderating factor. Third, the influence of external cues is substantial, with credibility endorsement demonstrating a second-order moderating effect (i.e., moderated moderation) on the impact of anthropomorphism. The details are listed in Table 6.

**Table 6:** Mechanisms of influence for different variables.

Conclusion	Key findings	Mechanism of influence
Human anchors have significant advantages in promoting policy adoption	Compared with digital human anchors, human anchors are more effective in enhancing the public’s willingness to adopt policies.	1. With their inherent social attributes and authenticity, human anchors are more capable of meeting the requirements of high-credibility information sources, thereby strengthening the persuasiveness of policy information itself. 2. Human anchors can narrow the psychological distance with the audience; the perception of psychological proximity can better promote the audience’s understanding, recognition of policy information, and ultimately their willingness to adopt the policy. 3. Psychological distance plays a key mediating role in the influence chain of “type of communication subject → willingness to adopt policies.”
The effectiveness of digital human anchors is situational	1. The effectiveness of digital human anchors is affected by the degree of anthropomorphism; a low degree leads to an increase in psychological distance. 2. Digital human anchors with a high degree of anthropomorphism can shorten the psychological distance and reduce cognitive load.	1. When the degree of anthropomorphism of digital human anchors is low, their “non-human” characteristics (such as technical traces and unnaturalness) are easily perceived by the audience, which widens the psychological distance—human anchors can more easily narrow the psychological distance. 2. When digital human anchors reach a high level of anthropomorphism through technological progress and can be perceived by the audience as “quasi-social actors,” they can effectively reduce the cognitive load and sense of alienation caused by their virtual nature.
Credibility endorsement has a moderating effect	1. Credibility endorsement significantly affects the roles of communication subject type and anthropomorphism degree on psychological distance. 2. Strong credibility endorsement can reduce the sensitivity to differences in the inherent characteristics of anchors.	1. In situations that lack strong endorsement, the audience is more likely to rely on the type of anchor and the degree of anthropomorphism to form judgments. 2. Strong credibility endorsement (such as authoritative certification) dominates the audience’s perception, enabling digital human anchors with high anthropomorphism to narrow the psychological distance with human anchors through external endorsement. 3. Even with strong endorsement, digital human anchors with low anthropomorphism may still not be able to fully overcome the disadvantage in psychological distance.

### 5.2 Theoretical contributions

Contextualized by the increasing integration of digital technology within public governance, this study systematically investigates the differential communicative efficacy of human versus digital anchors in policy dissemination and their underlying mechanisms of action. By employing a rigorous online experimental design, this study offers novel cognitive insights and empirical evidence for pertinent theoretical fields. The specific theoretical contributions of this study are as follows:

First, it significantly broadens the existing academic agenda by extending the investigation of digital communicators to the critical domain of public policy communication. Prior scholarship has predominantly explored digital human applications in commercial contexts, such as marketing (Han et al., 2023) and customer service or service recovery (Liu et al., 2023). Given the scarcity of current research focusing on the field of public policy communication, our findings not only corroborate the current general advantage of human anchors in fostering policy adoption intentions, but also reveal the potential for high-anthropomorphism digital anchors to mitigate this efficacy gap under specific circumstances, such as when paired with strong credibility endorsements. This contribution addresses a theoretical lacuna at the nexus of digital communication technology, public administration, and political communication while also providing crucial insights for delineating the appropriate application boundaries for digital humans in public affairs.

Second, this research enhances the comprehension of social cognitive mechanisms and the role of psychological distance in HCI. Moving beyond prior studies that often centered on specific emotional responses such as empathy (Liu-Thompkins et al., 2022), humor (Xu and Liu, 2022), or apology (Song et al., 2023), the current work concentrates on the pivotal dimension of perceived psychological distance toward different communicators. It confirms the critical mediating function of psychological distance in the relationship between communicator type and policy adoption intentions and innovatively reveals how the level of anthropomorphism moderates this psychological process. These findings indicate that high levels of anthropomorphism effectively reduce perceived psychological distance by closely simulating human characteristics. They advance anthropomorphism theory by emphasizing static feature descriptions to understand dynamic social perceptions in interactive contexts. Moreover, these results underscore anthropomorphism’s influence on superficial user experience and profound impacts on audience cognition, attitudes, and behavioral intentions, through the mechanism of reshaping psychological distance.

Furthermore, this study highlights the distinct value of credibility endorsement in modulating the efficacy of novel communicators in the digital era, and promotes the integration of relevant theories. The empirical results demonstrate that strong credibility endorsements significantly augment the communication effectiveness of digital human anchors, particularly those with high anthropomorphism. The underlying mechanism suggests that potent external credibility signals can mitigate or neutralize the inherent limitations of digital communicators concerning perceived authenticity and social proximity, thereby influencing the manifestation of anthropomorphic effects. This finding enriches the theoretical discourse on reputation governance in public management (Pedersen and Salomonsen, 2023), illustrates credibility endorsement as a crucial link among organizations, interactive technologies, and the public, and offers new perspectives for public sector credibility management amidst digital transformation. Concurrently, this study integrates and empirically validates the key tenets of persuasion theory within digital policy communication: communicator type (source attributes), anthropomorphism (message presentation style), and credibility endorsement (contextual cues) collectively influence audience attitudes and intentions. This integrated multifactorial approach advances the development of a comprehensive theoretical framework for understanding complex policy communication dynamics in the digital era, moving beyond single-factor analyses.

### 5.3 Management insights

This study’s findings not only enrich the relevant theoretical knowledge, but also offer targeted practical insights for the public sector to effectively leverage digital anchors for policy communication in an increasingly digitized environment.

1. Implementing differentiated deployment to establish a new framework for human-machine collaborative communication.

Research has confirmed that human anchors retain a broad advantage in fostering policy adoption, especially when deep trust and emotional connections need to be established. For major complex policies or those involving significant adjustments to public interests, senior human anchors should be prioritized for authoritative interpretation to leverage their unique values in professional expertise, empathy, and trust building. For routine, high-frequency tasks, such as information dissemination, standard Q&A, and service guidance, digital anchors (especially highly anthropomorphic types) can be deployed to enhance efficiency and coverage. In China, cities such as Shenzhen and Chengdu have achieved notable success in disseminating carbon neutrality and energy conservation policies through the introduction of digital hosts. More importantly, regions can advance human-machine collaboration in tiers based on their economic and technological capabilities. For instance, resource-rich central cities can adopt a “human-led, digital-assisted” model for major policy announcements and complex issue communications, with human anchors taking the lead in in-depth explanations. Concurrently, digital anchors provide multimodal background materials, terminology explanations, or real-time interactive Q&A to enhance credibility. This complementary approach transforms communication from “broad coverage” to “targeted precision,” to achieve synergistic dissemination.

2. Optimizing anthropomorphic design to reduce digital anchors’ psychological distance.

This study highlights the critical role of anthropomorphism in regulating the effectiveness of digital anchors, with highly anthropomorphic ones significantly narrowing the psychological distance between them and their audiences. Therefore, the practical application of digital human anchors should not merely aim for superficial resemblance, but also pursue a deeper sense of authenticity. Resources must be invested in continually enhancing the multimodal expressiveness of digital anchors, including more natural speech intonation, richer facial expressions, and more coordinated body movements. Additionally, exploring the integration of affective computing technology is essential for endowing them with situational awareness and emotional interaction capabilities. The goal is to make digital hosts perceive themselves as “socially acting entities” rather than as cold machines, thereby effectively reducing psychological barriers among the public and enhancing the smoothness and receptiveness of information delivery.

3. Utilizing authoritative endorsements to bolster digital anchor credibility while guarding against “labeling” risks.

A key finding of this study is the significant second-order moderating effect of strong credibility endorsement, which effectively narrows the perceived psychological distance gap between high-anthropomorphism digital and human anchors. This effect underscores the critical importance of prioritizing and effectively leveraging credibility endorsements when promoting the use of digital avatars for policy communication. Public sector entities must provide clear and authoritative identity verification and source attributes for digital avatars (Li and Yang, 2024). For instance, they can be integrated into official media platforms or government portals through endorsements or certifications from the relevant authoritative bodies. Such robust external signals can significantly enhance the credibility of digital hosts, compensating for their inherent limitations in authenticity and social presence, thereby increasing public trust in and acceptance of the information they convey. However, strong credibility endorsements are merely a “steppingstone” for establishing initial public trust. Only through professional cultivation of knowledge dissemination, emotional interaction, and value expression can digital hosts better showcase the unique personality and value of this new communication entity. These factors enable the public to “trust because of the content” rather than “trust because of authority,” achieving the leap from being trusted to being trustworthy.

4. Establishing robust governance and ethical norms for digital human applications.

Optimizing “anthropomorphic design” and effectively leveraging “authoritative credibility endorsements” as key strategies to enhance the communication effectiveness of digital avatars carry potential ethical risks and governance challenges. Therefore, establishing robust governance standards are necessary and urgent. To ensure the responsible application of digital avatars in policy communication, comprehensive technical standards (Batool et al., 2025), application scenarios (Park, 2024), and guiding principles or norms for ethical constraints (García de Torres et al., 2025) should be promptly formulated. Among these, an ethical responsibility exists to safeguard the public’s right to know through mandatory, clear “AI-generated” identity markers (Veale and Zuiderveen Borgesius, 2021). Avoiding confusion among real individuals is fundamental to maintaining transparency and securing the public’s right to know. Simultaneously, clearly defining appropriate application scenarios (such as policy interpretation and public services), strictly prohibiting overreach, and establishing content reviews and public feedback mechanisms are essential for mitigating risks, facilitating continual improvement, and fostering public trust (Yuan and Chen, 2025).

## 6 Research limitations and perspectives

Although this study produced preliminary findings, it had several limitations that highlight directions for future research.

First, the research focus and sample representativeness were limited. Primarily, the study centered on the specific theme of “plastic pollution control,” and the applicability of its conclusions to different policy domains such as healthcare and education remains to be tested. Second, although this study excluded student groups, the sample primarily concentrated on specific online communities. The origin and scale of these groups do not fully represent the broader public landscape. Public acceptance of digital avatars varies across regions, age groups, and occupations. Third, the manipulation and measurement of “anthropomorphism level” were overly singular. This study primarily manipulated “anthropomorphism level” through variations in visual appearance, failing to systematically incorporate multimodal cues such as voice (e.g., tone and intonation) and linguistic style (e.g., formal vs. colloquial). Fourth, insufficient attention has been paid to differences in individual audience characteristics. This study did not sufficiently examine the cognitive differences stemming from the cultural and linguistic backgrounds of the audience. Furthermore, the impact of individual psychological traits, such as political trust, media literacy, and digital affinity, on the acceptance levels of digital human anchors warrants further exploration.

To address these limitations, future research should broaden the diversity of research contexts and samples. They should extend experimental settings to more diverse policy domains, such as healthcare and educational reform, while striving to expand sample coverage through cross-regional sampling (urban and rural populations) to enhance the external validity of the conclusions. Furthermore, future studies should deepen the multimodal manipulation of “anthropomorphism levels.” They should move beyond the singular visual dimension to systematically examine the independent or interactive effects of multimodal dimensions, such as voice pitch, vocal affect, and linguistic style, on digital avatar anchors. This expansion provides more concrete empirical guidance for design optimization. Moreover, the research should integrate individual audience characteristics into a comprehensive analysis. Cross-cultural comparative studies can test the applicability of this study’s findings across different cultural and linguistic contexts. Key individual difference variables—such as audience political trust, media literacy, and digital affinity—can be systematically incorporated into research models as moderators or covariates. This approach should reveal attitude differences among distinct audience segments toward digital anchors and their underlying mechanisms.

## Figures and Tables

**Figure A1:**
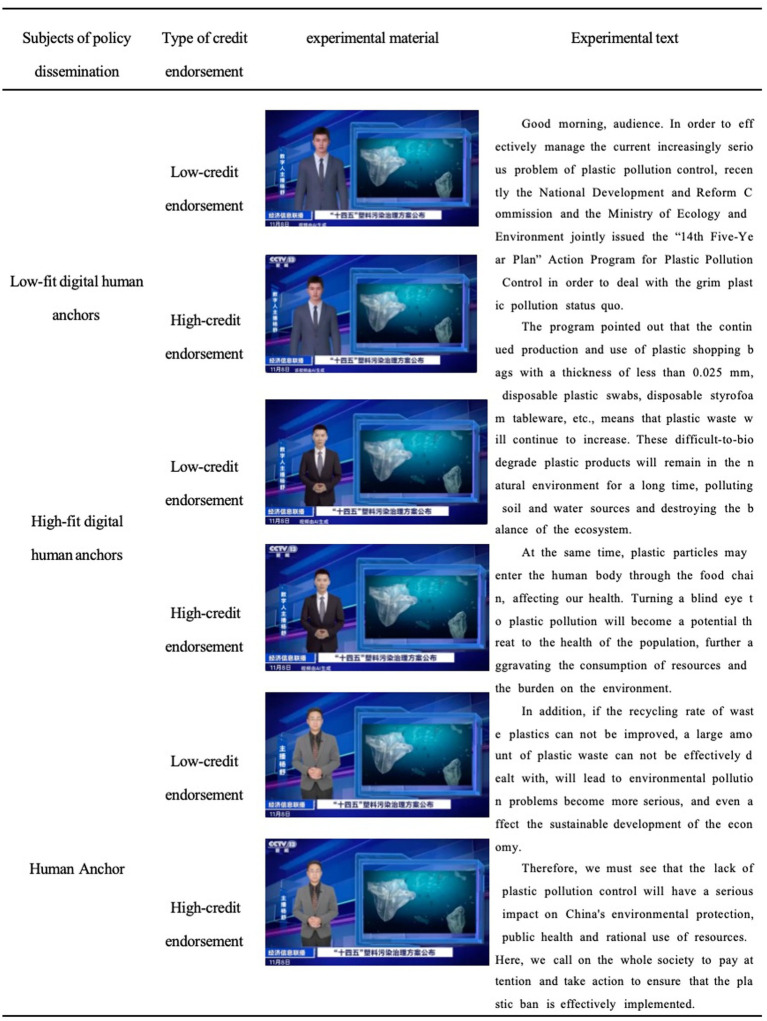
Description of experimental materials.

## Data Availability

The original contributions presented in the study are included in the article/supplementary material, further inquiries can be directed to the corresponding author.
